# The Great Impostor Did It Again: Syphilitic Arthritis

**DOI:** 10.7759/cureus.17344

**Published:** 2021-08-21

**Authors:** Xue Ao, Jia Hong Chen, Priyaranjan Kata, Anish Kanukuntla, Veera Bommu, Michael Rothberg, Pramil Cheriyath

**Affiliations:** 1 Internal Medicine, Hackensack Meridian Ocean Medical Center, Brick, USA

**Keywords:** syphilitic arthritis, syphilis, congenital syphilis, tertiary syphilis, polyarthritis, sexually transmitted infection, penicillin g

## Abstract

Syphilis-related bone and joint involvement is commonly found in congenital form, but it can also be seen in adults with acquired syphilis as a rare sequela of infectious syphilis. We report a case of syphilitic arthritis where the patient presented with multiple problems over the course of several visits and was eventually diagnosed with tertiary syphilis as the source of his musculoskeletal complaints. The clinical manifestations of syphilis can be diverse and challenging, as evidenced by our case. Unusual clinical manifestations might be seen in syphilis, and clinicians may not be familiar with these clinical presentations while diagnosing. Being aware of arthropathy in syphilis and including it in the differential diagnoses will help improve patient outcomes and avoid unfavorable consequences, particularly in the high-risk group.

## Introduction

Syphilis is a commonly encountered infection worldwide, with an annual incidence of approximately 10-12 million cases [1]. It is a sexually transmitted disease caused by *Treponema pallidum*, a spiral-shaped bacterium. Syphilis has multisystemic involvement including skin and cardiovascular and central nervous systems; however, musculoskeletal system involvement is an unusual presentation and is rarely reported. Syphilitic arthritis is seen in congenital and tertiary syphilis. The incidence of syphilis has been increasing and the highest number of cases since 1991 was reported in 2018: more than 35,000 cases. The number of cases of congenital syphilis has increased by 40% and so has the number of cases of syphilitic arthritis [2]. In this report, we present a rare case of syphilitic arthritis in a 44-year-old male who responded well to treatment with penicillin.

## Case presentation

A 44-year-old male presented to the emergency room with left shoulder pain of one day's duration. He also had fatigue, cough, arthralgias, and joint swelling. On physical examination, he was found to have decreased abduction of the left shoulder due to pain, and pain in the left trapezius with shoulder movement. The X-ray of the shoulder was unremarkable. All the labs were unremarkable including the Lyme disease panel. The patient was discharged on pain medication.

Three months later, the patient presented to the ER with complaints of right-sided flank pain of one day's duration. The review of systems and physical exams were unremarkable. Labs were notable for small amounts of blood in urine analysis but were otherwise within normal limits. The CT scan of the back at that time showed no evidence of acute pathology. The patient was given IV fluids and pain control medication. His condition improved and he was discharged.

**Figure 1:**
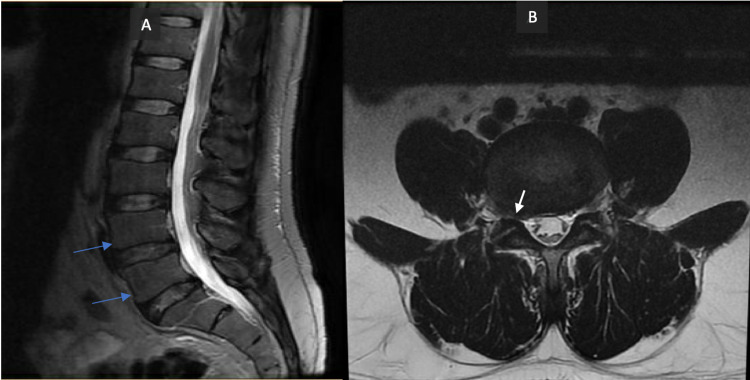
MRI of the lumbar spine (A): mild multilevel degenerative changes (blue arrow) on the sagittal plane and (B) complex asymmetric to the right with the mild facet arthropathy and ligamentum flavum hypertrophy causing moderate right neural foraminal stenosis (white arrow); mild to moderate left neural foraminal stenosis on the axial plane MRI: magnetic resonance imaging

Three days later, he returned to the ER with sharp back pain that he rated as a 10 out of 10 on the pain scale. He could not walk due to back pain. Vital signs were within normal limits. A review of systems was positive for left shoulder pain. On physical examination, he had a pruritic rash on the neck, and his leg strength was a 3/5 on neurological examination. He claimed to be in a monogamous relationship when questioned about his sexual history. All labs were within normal limits, and a CT scan of the head and lumbar spine revealed no abnormalities. MRI of the lumbar spine showed mild degenerative changes and ligamentum flavum hypertrophy, resulting in moderate neural foraminal stenosis (Figure 1). He was admitted for foraminal stenosis and treated with steroids, and his condition subsequently improved. He was discharged three days later.

The patient went to visit his primary care doctor within a week. He was tired and could not feel his nose or ears. He had urinary incontinence and felt as if he was out of his body. His primary physician ordered a complete blood count (CBC), comprehensive metabolic panel (CMP), thyroid screen, tick panel, sexually transmitted infection (STI) workup, erythrocyte sedimentation rate (ESR) test, and a vasculitis workup. STI workup was positive for rapid plasma reagin (RPR) and venereal disease research laboratory (VDRL) test. When the patient returned the next week, he experienced myalgia and was disoriented. A lumbar puncture was performed, which was positive for syphilis. He admitted to having a sexual encounter four months ago and to periodically having genital warts that usually resolved after two or three days. He was admitted to the hospital and treated with penicillin G potassium four million units intravenously every four hours for 14 days. He responded well to penicillin and was discharged home without any complications.

## Discussion

*Treponema pallidum* is thought to enter through the areas of microtrauma, often from mucous membranes. Syphilis has two phases; the first phase is the early infectious one and the next is the late non-infective phase [3]. There are four stages of syphilis with different clinical presentations in each stage: primary (local), secondary (generalized), latent (asymptomatic), and tertiary (cardiovascular, neuro, gummatous) syphilis. Rheumatic clinics reported a fresh increase in the number of cases of syphilitic arthritis in 1963, a few years after the Second World War, when syphilis was not adequately treated and resulted in an increased number of congenital syphilis cases [4].

*Treponema pallidum* spreads hematologically to the metaphyseal region (which has a rich blood supply) and enters medullary cavities of bones, inducing the inflammatory response by lymphocytes and plasma cells. Usually, if there is adequate defense, it will get healed with fibrosis. However, the persistence of bacteria destroys the tissue and causes necrosis resulting in arthritis and/or polyarthralgia. A few patients with tabes dorsalis can develop neuropathic arthropathy as a late complication. Syphilitic arthritis presents as marked swelling, tenderness, and restricted range of motion in the affected joints. Polyarthritis with synovitis is associated with tertiary syphilis. Synovitis is the primary reason for rheumatic features in syphilis and usually presents as migratory polyarthralgia. The most commonly involved joints are bilateral knees, hips, shoulders, and proximal interphalangeal joints, and it presents as subacute to chronic disease. A few patients might present with tenosynovitis of the hands. Pain is usually most severe at night, aggravated by heat, and usually relieved by movement [5]. Congenital syphilitic arthritis is classified by D'Arcy Power into Clutton's joints, chondro-arthritis, suppurative arthritis, gummatous synovitis, hydrarthrosis, symmetrical serous synovitis, ulcerating, or Von Gie joints. Axhausen described two forms of tertiary syphilitic arthritis in 1914: osseous and synovial. The synovial form is seen mostly in children and occurs as perisynovitis, which is characterized by inflammation around the synovial membrane. On the other hand, the osseous form, which is commonly seen in young patients, presents as epiphysitis; eroded and irregular articular surface, and cavitation in the interior of the bone, along with bony outgrowths. In adults, one whole large joint might get affected with osteoarthritic features [4]. Less frequently, migratory polyarthralgia or arthritis can occur in secondary syphilis, which might resemble the presentation of rheumatic fever.

The exact diagnosis of syphilitic arthritis depends on multiple factors including other clinical and radiological manifestations of congenital syphilis, family history, and therapeutic response to anti-syphilitic medications. X-ray of Clutton's joints reveals effusion without bone involvement. Gummatous synovitis does not have bone involvement; however, they can develop disuse osteoporosis as a result of the patient's pain. The Von Gie joint has reduced joint space and well-demarcated bone erosions towards the central areas of the joint that are evidenced radiologically [4]. Synovial fluid analysis in the affected patients has revealed polymorphonuclear neutrophils and phagocytic mononuclear cells with large vacuoles when visualized under electron microscopy. Although spirochetes were not directly visualized in synovial fluid analysis, the non-migratory character of arthritis and association with the characteristic mucocutaneous features of secondary syphilis can help in the diagnosis [6]. Immunofluorescent anti-treponemal antibody absorption test reveals spirochetes in joints. Our patient presented multiple times with polyarthralgia (shoulder, back) and other non-specific features like fatigue. X-ray of the shoulder and CT scan of the lumbar spine did not reveal any abnormalities. MRI spine revealed mild degenerative changes and foraminal stenosis.

The earlier the syphilitic arthritis is diagnosed and treated, the better the prognosis. Syphilitic arthritis is treated with intravenous injection of benzathine penicillin G potassium four million units every four hours for 14 days. In addition to this, local management of the joints with massage and physiotherapy is also important to prevent ankylosis of the joint [4]. Pregnant women with syphilis should be treated to prevent congenital syphilitic arthritis. They should be treated with penicillin and if they are allergic to penicillin, then they need to get desensitized before treatment. Patients who have had syphilis must be followed up regularly after treatment, as relapse is common and can be asymptomatic [7]. A large prospective randomized trial has suggested that 2 g oral azithromycin is equally effective in treating early syphilis as benzathine penicillin, which would be a major advancement in syphilis control [8]. Ankylosis is a common complication of untreated syphilitic arthritis, which results from destructed articular cartilage causing gross deformity with severe crippling. Consequently, an ankylosed joint impedes the freedom of movement and stability and is more prone to trauma.

Our patient was initially diagnosed with foraminal stenosis and he responded well to corticosteroids. But the recurrence of arthralgia and worsened condition with disorientation prompted us to perform a lumbar puncture, cerebrospinal fluid (CSF) analysis, and an STI workup that revealed syphilis. He was eventually diagnosed with syphilitic arthritis, which was due to tertiary syphilis. This was evidenced by his positive sexual history and positive VDRL test in CSF and improvement of symptoms after treatment with penicillin G. We were able to prevent the debilitating complications of syphilitic arthritis such as ankylosis in our patient thanks to early diagnosis and prompt treatment.

## Conclusions

Syphilitic arthritis is one of the unusual presentations of syphilis. Syphilis can remain latent until it reaches the tertiary stage, at which point syphilitic arthritis manifests. Family history and sexual history are the keys to identifying patients at high risk. Primary care physicians and orthopedic surgeons should be aware of syphilitic arthritis and keep it in their differential diagnoses, so that appropriate and early diagnosis can be made and the condition can be promptly treated to prevent long-term morbidity.
